# Democratic Population Decisions Result in Robust Policy-Gradient Learning: A Parametric Study with GPU Simulations

**DOI:** 10.1371/journal.pone.0018539

**Published:** 2011-05-04

**Authors:** Paul Richmond, Lars Buesing, Michele Giugliano, Eleni Vasilaki

**Affiliations:** 1 Department of Computer Science, University of Sheffield, Sheffield, United Kingdom; 2 Gatsby Computational Neuroscience Unit, University College London, London, United Kingdom; 3 Department of Biomedical Science, University of Antwerp, Wilrijk, Belgium; The University of Western Ontario, Canada

## Abstract

High performance computing on the Graphics Processing Unit (GPU) is an emerging field driven by the promise of high computational power at a low cost. However, GPU programming is a non-trivial task and moreover architectural limitations raise the question of whether investing effort in this direction may be worthwhile. In this work, we use GPU programming to simulate a two-layer network of Integrate-and-Fire neurons with varying degrees of recurrent connectivity and investigate its ability to learn a simplified navigation task using a policy-gradient learning rule stemming from Reinforcement Learning. The purpose of this paper is twofold. First, we want to support the use of GPUs in the field of Computational Neuroscience. Second, using GPU computing power, we investigate the conditions under which the said architecture and learning rule demonstrate best performance. Our work indicates that networks featuring strong Mexican-Hat-shaped recurrent connections in the top layer, where decision making is governed by the formation of a stable activity bump in the neural population (a “non-democratic” mechanism), achieve mediocre learning results at best. In absence of recurrent connections, where all neurons “vote” independently (“democratic”) for a decision via population vector readout, the task is generally learned better and more robustly. Our study would have been extremely difficult on a desktop computer without the use of GPU programming. We present the routines developed for this purpose and show that a speed improvement of 5x up to 42x is provided versus optimised Python code. The higher speed is achieved when we exploit the parallelism of the GPU in the search of learning parameters. This suggests that efficient GPU programming can significantly reduce the time needed for simulating networks of spiking neurons, particularly when multiple parameter configurations are investigated.

## Introduction

As the bounds of single processor speed-up have reached a stringent limit, the self-fulfilling “Moores Law” dictating a doubling of computational speed roughly every 24 months can only be realised by increasing the number of processing cores on a single chip. Inevitably this has serious implications on the design of algorithms that must take into account the resultant parallel architectures (parallelisation). Similar to multi-core CPU systems, the Graphics Processing Unit (GPU) is a parallel architecture which is currently emerging as an affordable supercomputing alternative to high performance computer grids. In contrast to multi-core parallelism, the GPU architecture consists of a much larger number of simplistic vector processing units which follow a stream-like programming model [1], [2]. The availability of high quality GPU programming tool-kits such as the Compute Unified Device Architecture (CUDA) and Open Computing Language (OpenCL), has without doubt propelled GPU computing into the mainstream. Despite this, GPU programming requires careful optimisations and knowledge of the underlying architecture in order to gain notable performance speed-ups. It is therefore imperative to use only algorithms which form a good fit to GPU hardware by exploiting large amounts of fine grained parallelism when applying GPU programming to scientific problems such as the simulation of populations of biologically plausible neurons which we explore in this paper.

The purpose of this work is twofold. First, we demonstrate that GPU can be efficiently used in the context of Computational Neuroscience, as a low cost alternative to computer clusters. Second, using the GPU computing power, we study how specific network architectures of biologically plausible (spiking) neurons perform in learning an association task. More specifically, we simulate a two layer network of spiking neurons entirely on the GPU. The input layer represents the location of an artificial animal and the output layer its decision, i.e. which action to perform. We investigate two alternative architectures. In the first, the output layer has recurrent, “Mexican-Hat”-type connectivity[3]–[5], i.e. short range excitatory, long range inhibitory connections. This type of connectivity has been identified in cortex organisation [6]–[8] and offers a “plausible” neural network implementation for reading out the information (the decision) encoded in the output layer. We term this scenario “non-democratic” decision making, as the participation of the neurons in the decision is influenced by others via recurrent (lateral) connections. As a consequence, first occurring spikes can significantly affect the activity bump formation in the recurrent network. In the second architecture there are no recurrent connections. The readout of the encoded information in the output layer is done via a “population vector” [7], [9], without taking into account how this could be implemented in neural networks terms. We term this scenario “democratic” decision making, as every neuron participates in the decision without being influenced by others. Learning in both scenarios takes place by modifying the synapses between the input and the output layer according to a spike-based Reinforcement Learning rule derived from reward optimisation by gradient ascent [10]–[12], an approach that can be linked to policy gradient methods in machine learning [13], [14].

Our simulations indicate that “non-democratic” decision making, which is based on “Mexican-Hat”-type connectivity, is prone to “crosstalk” of learning when input-layer neurons are participating in the representation of multiple states. In our setup, this is due to overlapping neuronal receptive fields. We borrow the term “crosstalk” from the field of electronics in the following sense. If a neuron in the input layer is active during more than one state (network input), it can be considered as part of more than one sub-network. Synaptic changes that take place in one sub-network involving this specific neuron may affect the output of another sub-network, inducing therefore “noise” in the decision making process. The advantage of the “democratic” decision making over the “non-democratic” is that “crosstalk” tends to cancel out due to the linear nature of the population vector readout and the symmetry of the task. We further underline this argument with sets of experiments, which investigate the influence of varying the reward function and the effect of additive noise. The results of the study are presented in section **Results**.

The simulations presented here would have been extremely time consuming (or even virtually impossible) on a low cost desktop computer without the use of GPU programming. Our GPU simulations shows speed-ups of up to a factor of 42 compared to a serial implementation running on a Intel i7-930 quad core CPU. Our implementation is also parallel over a number of independent simulations, enabling us to produce statistically accurate results and also to perform rapid searches over various parameter configurations. This functionality has allowed us to scan vast parameter spaces demonstrating important and general differences between the two systems of “democratic” and “non-democratic” decision making. The code developed is presented in **Methods** together with a brief introduction to GPU Programming and a discussion of the resulting performance gain and future work.

## Results

In this section, we introduce and signify the importance of the modelling problem, which is an essential element of a navigation scenario. We describe the network architecture in detail and present the simulation results for various configurations. Finally, we draw conclusions related to the performance of the learning system. More specifically, when the neurons in the output layer do not equally contribute to the decision taken, but are influencing each other via strong, non-linear dynamics, the system is more susceptible to noise and learning performance generally degrades.

### The Simulation Paradigm

Action selection mechanisms are often modelled as “winner-take all” or “winner-take most” networks. Making a decision involves some kind of competition [15], [16] where the winner exhibits maximum activation and hence represents the decision whereas the losers' activity decays to a low state. These mechanisms, that are typically modelled by lateral connectivity, are not only attractive from a conceptual point of view but are scenarios worthy of exploration when building models, as they can provide simple mechanisms by which information can be sent to other neurons. Evidence of short range excitatory/long range inhibitory connectivity has been identified in cortex organisation, see for instance [6]–[8] and references therein.

Decision making [17]–[21] can be also considered in the context of Reinforcement Learning [22]–[24], where an agent explores its environment and learns to perform the right actions that bring maximal reward. Such scenarios are often incorporated in behavioural animal studies, where, for instance rodents are learning to perform specific tasks that involve spatial learning memory. One such task is the Morris water-maze[25].

A previous model [12] studied a Morris water-maze navigation scenario, similar to [26]–[30], but implementing the agent with a spiking neural network. This specific model explored “Mexican-Hat”- type lateral connectivity (MHC) as a simple “biologically plausible scenario” for reading out information and action selection. In fact, neurons do not simultaneously form excitatory and inhibitory synapses, but such a behaviour could in principle be achieved via interneurons. The MHC introduces a non-linear (non-democratic) effect in the decision making process, as all neurons are silenced by the inhibition introduced by the MHC except for a small group of neighbouring neurons which form an “activity bump”. This bump can be interpreted as corresponding to the winning action (i.e. the animal decision), suggesting a simple mechanism to extract information out of a competing group of neurons, that can be implemented easily in neural networks, without the need of additional “plugged on” read-out mechanisms.

Learning in this network model takes place by modifying the synapses via a spike-based Reinforcement Learning rule [12] derived from reward optimisation by gradient ascent [10], [11], [31]–[35], an approach that can be linked to policy gradient methods in machine learning [13], [14]. Other classes of spike-based Reinforcement Learning rules are based on Temporal-Difference (TD) learning [36]–[39], in particular actor-critic models [23], [37], [40] and on the extension of classical STDP models [41]–[43] to reward-modulated STDP rules [11], [44]–[46].

Interestingly, learning was shown to fail when MHC-based architectures were combined with the policy gradient spiking Reinforcement Learning rule derived in [10]–[12] (within the range of tested parameters). If however, we are not concerned about how the population decision is communicated between layers of neurons (and therefore do not implement MHC) but simply read out the activity via the population vector [7], [9], learning does not fail. We term this operation a“democratic decision”, because the non-linear effect of the lateral connections is absent from the process: all neurons “vote” freely without being “influenced” by other neurons and their votes are weighted by their activity (in a linear fashion) so every spike per neuron counts the same.

These findings naturally raise the following questions. Which precisely are the conditions that favour the “democratic” decisions versus the “non-democratic” ones? In order to answer this question, we study the results of a similar problem but with a reduced complexity (in one dimension) that allows a systematic parameter study via high performance GPU simulation. Our animat (artificial animal) “lives” on a circle (1D space) and performs the following task. We set the animat randomly to one position on the circle (encoded as an angle from 0 to 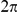). The animat then chooses a direction. At each position there is one “correct” direction, which depends smoothly on the position; if the animat chooses it, it will receive maximum reward. Choices “near” the correct direction also receive some reward, according to a reward function. This smoothness assumption with respect to the rewarded action makes learning possible. The setting of the animat at a location and selection of a decision constitutes a single trial. After completion of a trial the animat is placed randomly at a new position and the task is repeated. The problem will be fully learned if the animat chooses the correct direction at each position in the cycle. In the simulations that follow, without loss of generality we assume that the “correct” (maximally rewarded) direction is the one that is equal to the initial position that the animat is placed.

### Model Architecture

**Figure 1:**
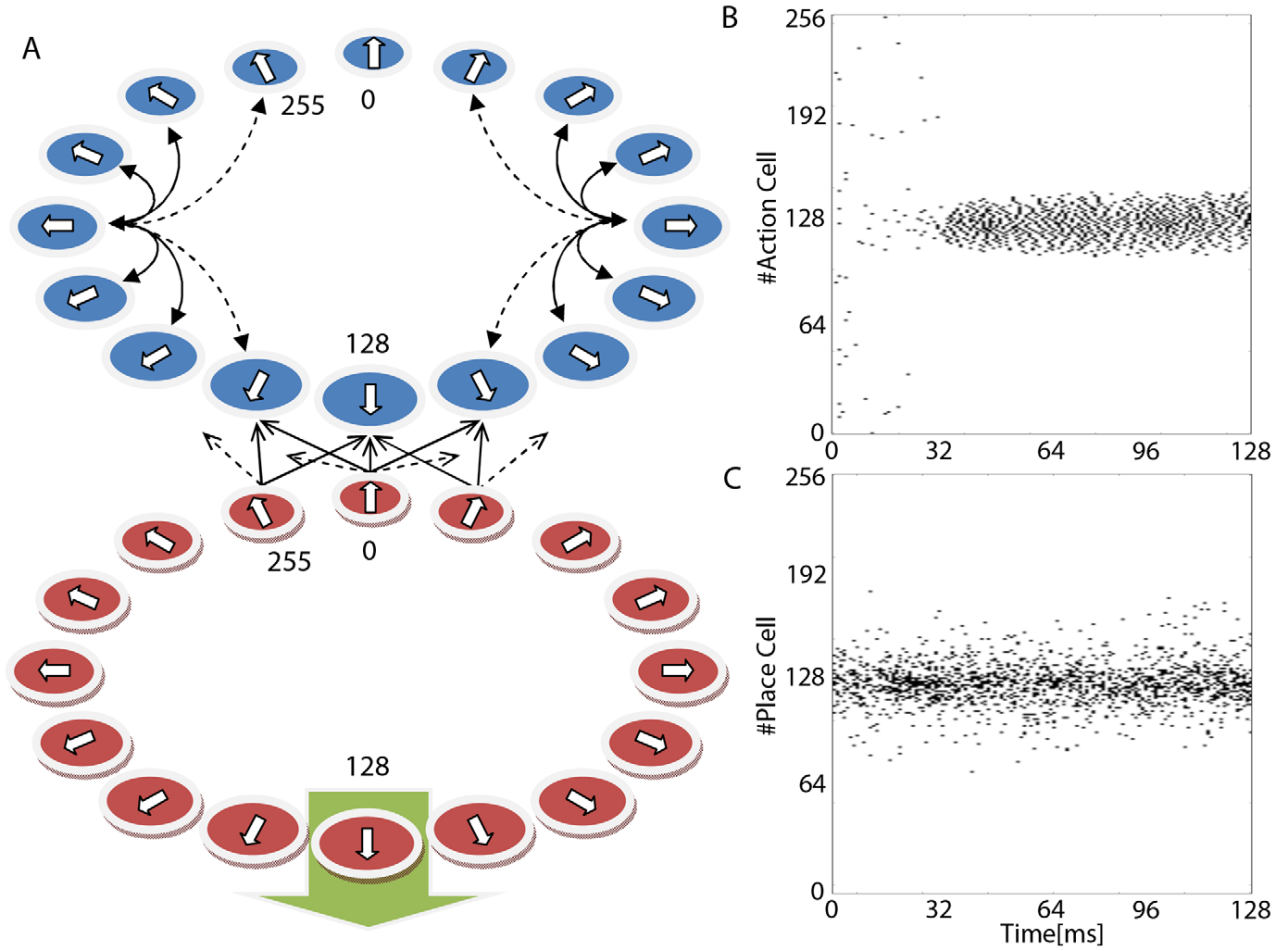
Model Architecture. Our animat (artificial animal) “lives” on a circle and performs the following task. We place the animat randomly to one position on the circle. The animat then chooses a direction, the decision, 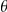. At each position there is one “correct” direction 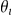. Choices 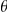 close to the correct direction 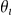 receive some reward, according to a Gaussian reward function. This processes (the setting of the animat at a location, selection of a decision, receiving a reward and updating of the feed-forward weights) constitutes a single trial. After completion of a trial the animat is placed randomly at a new position and the task is repeated. The task will be fully learned if the animat chooses the correct direction at each position on the circle. A: Shows a schematic overview of our two layer model architecture consisting of Place Cells (red) and Action Cells (blue). Place Cells (modelled as Poisson neurons) are connected to Action Cells (Integrate-and-Fire neurons) using an all-to-all feed forward network (not all connections are shown). In addition Action Cells may be interconnected via lateral Mexican hat-type connections (not all connections are shown). The layer of Place Cells is arranged in a ring like topology with each neuron 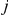 having a preferred angle, and firing with maximum probability if the location of the animat happens to coincide with this preferred angle. In the example shown the animat is placed at the location that corresponds to the preferred direction of neuron index 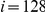. The top layer, also arranged in a ring topology, codes for the location the animat will choose. B: Shows the output spike train of the Action Cells demonstrating a bump formation around neuron (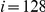) with a resulting decision angle 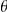 matching the preferred angle of 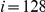. In this example the target angle 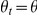, and therefore the animat has made the correct decision. C: Shows the spike train of the input layer (Place Cells) when the animat is placed at the location encoded by neuron 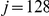.

Our model architecture implements a simple two-layer network of neurons. The cells of the input layer, which we term “Place Cells” due to the conceptual similarity to the biological place cells discovered by O'Keefe and Dostrovsky in 1971 [47], are arranged on a circle. Each cell has a tuning curve with a unique preferred direction (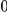 to 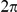). Preferred directions are equally spaced on the circle. In accordance with evidence [48], the ensemble of Place Cells codes for the position of the animat. The output layer has a similar structure; each output cell has a preferred direction and the population vector of the ensemble codes for the “action” the animat takes. A schematic diagram of the network is shown in Figure 1A.

We study two variants of the same architecture, one with a recurrent Mexican-Hat connectivity (MHC) among the neurons of the output layer, and one without lateral connections. We note that in the case where MHC is present, the population vector, which decodes the decision, points very precisely at the direction that corresponds to the neuron(s) with the maximal activity. For a direct comparison, we use the population vector in both architectures to extract the decision of the output cells, though it would have being equivalent if, in the case of lateral connections, we were reading out the decision as the activity peak.

In Figure 1B we show the activity of the input layer, the Place Cells. In Figure 1C we plot the activity of the output layer (“Action Cells”) for the network with MHC. Learning is achieved by the modification of the feedforward connections from the input layer to the output layer according to a Reinforcement Learning policy gradient spiking rule [10]–[12].

#### Place Cells

Place Cells are modelled as Poisson neurons. The stochastic firing rate 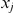 of Place Cell 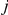 is determined by the distance between its preferred direction 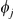 and the animat's location 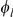:
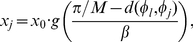
(1)where the function 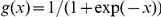 is the sigmoidal (logistic) function (the neuron's response function), 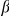 a positive parameter that determines the steepness of the sigmoidal function, 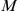 is the total number of Place Cells, and 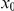 a factor that scales the maximum frequency at which the Place Cells can fire. The purpose of 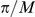 in Equation 1 is that the firing rate of neuron 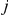 drops to half of the maximum (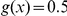) when the animat is placed in between neuron 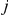 and either of its directly neighbouring neurons 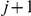, 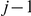, i.e. 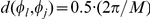. Parameter 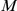 allows scaling of this property with the number of neurons. The distance 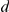 between the two angles 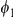 and 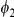 and is given by:
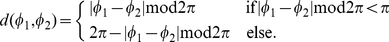
(2)


The neurons are Poisson for the following reason. Given the rate 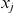 (which is constant in each trial), the spikes are generated according to a Poisson process with intensity 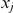, which is equivalent to saying that all spikes are independent with the expected number of spikes in an interval of length 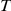 being 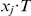. Throughout our simulations we use the constant values of 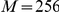, 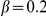 and 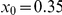 (unless otherwise stated) resulting in a maximum firing rate of 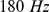 at the most active neuron in the population.

The parameter 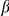 is crucial for the system as it determines the overlap of neighbouring receptive fields of the Place Cells. In plain words, every cell 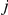 fires maximally when the animat is at its preferred location (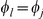). By making the sigmoidal function less steep (decreasing 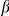), the neuron will respond with a higher probability when the animat is located far away from 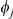. This results in that neuron 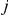 will contribute with a higher probability to the representation of more potential locations of the animat.

#### Action Cells

Action cells are modeled as Leaky Integrate-and-Fire units [49] with escape noise, which are a special case of the Spike Response Model [50]. The change in membrane potential 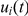 of a neuron 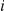, which receives input from Place Cell 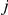 at time 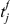 (with 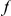 being an index on the individual spikes of neuron 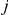), and input via lateral connections from Action Cell 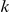 at time 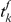, is given by:
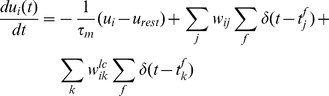
(3)where 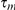 is the membrane time constant of 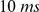, 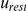 is the resting potential of 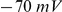 and 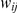 is the synaptic strength between the presynaptic (place) cell 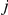 and the postsynaptic (action) cell 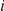. Furthermore 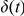 denotes the Dirac function and 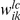 the synaptic strength of the lateral connection between Action Cells 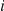 and 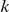.

Spikes are generated with an instantaneous probability 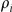 determined by an exponential function of the form:
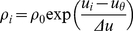
(4)where 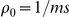 is a scaling factor of the firing rate. The parameter 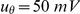 can be considered as a “soft” firing threshold and 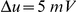 controls the "sharpness" of the “soft” threshold, see also [50], chapter 5.3.

After a neuron fires, its membrane voltage does not return directly to the resting potential. Instead, it increases its negative polarization even further (known as hyperpolarizing spike afterpotential). To account for this phenomenon, right after a spike is emitted we set the neuron's membrane potential to 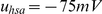, i.e. 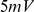 below the resting potential.

#### Lateral Connections

**Figure 2 pone-0018539-g002:**
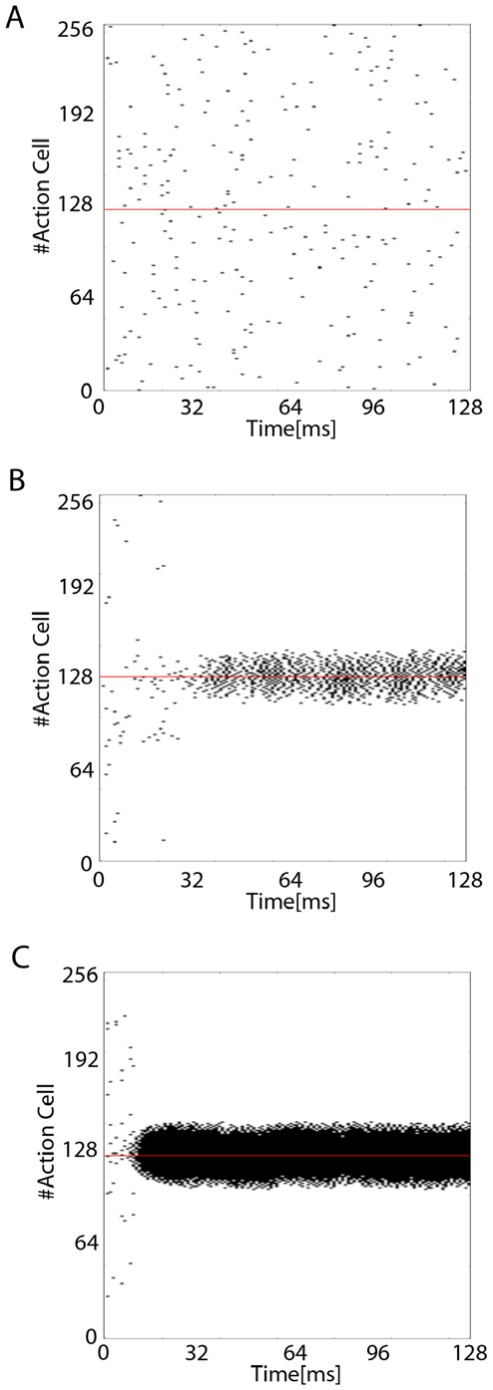
Lateral Connection Strength. Figure shows the effect of varying the lateral connection strength parameter 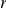 on the spiking activity of the Action Cells over a period of 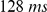 (before learning has taken place). For clarity, the figures show a decision angle of value of 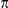 suggesting a centralised high activity around Place Cell index 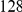 (as in Figure 1). A: Shows a system without lateral connections where 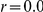. B: Shows a system with lateral connections where 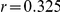. C: Shows a system with very strong lateral connections where 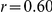.

In our model, the weights of the lateral connections are determined by a Mexican-Hat-shaped function of the distance between Action Cells 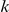 and 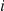, yielding a (fixed) synaptic strength 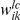 determined by:
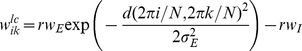
(5)where 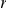 is the scaling of the MHC, 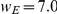 is the strength of the excitatory MHC weights, 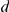 is the distance function defined in Equation 2, 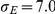 is the length scale of the MHC, 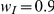 is the strength of inhibitory MHC, and 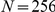 is the number of Action Cells. Parameter 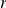 takes the value of 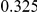 unless otherwise stated. In Figure 2 we plot the activity of the output layer for a system without lateral connections (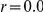, panel A), a system with lateral connections (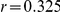, panel B) and a system with very strong lateral connections (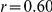, panel C).

#### Action Selection and Reward

For each cycle of the simulation the decision angle 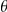 is determined from the average population vector of the neuronal activity over the cycle time 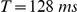 as follows:
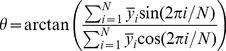
(6)where 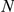 is the number of Action Cells and 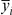 is the average neuronal activity of the Action Cell 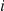 over the cycle calculated as:
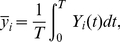
(7)with 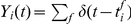 being the entire postsynaptic spike train of the Action Cell 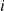 fired at times 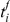.

Given the decision angle, a reward is determined by the following reward function:
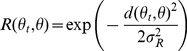
(8)where 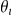 denotes the target angle, 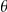 is the decision angle of the Action Cell population as determined by Equation 6, 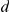 is the distance function defined in Equation 2 and 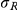 is the standard deviation of the reward function with a default value of 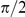 (unless specified otherwise in the text).

#### Learning Rule

Learning takes place by modifying the feedforward connections 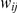 between place and Action Cells at the end of each trial according to the following policy gradient learning rule [10]–[12]:
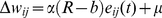
(9)where 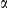 is the learning rate, 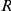 the reward calculated from Equation 8, 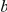 a reward baseline and 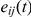 the eligibility trace [44] defined below. The variable 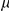 is a uniformly distributed random noise sample from the interval 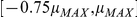. The reward baseline takes the value of 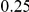 and its presence speeds up learning for both systems.

The eligibility trace 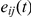 is a memory of the previous activity of the synapse, and is calculated as:
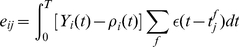
(10)where 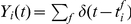 is the spike train of the postsynaptic neuron 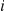, 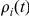 the instantaneous probability of firing and 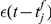 the time course of the excitatory (or inhibitory) postsynaptic potential (EPSP or IPSP) caused by the 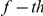 firing of neuron 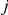, which is modelled as:
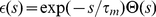
(11)with 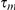 being the membrane time constant of 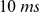 and 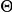 the step (Heaviside) function.

We would like to emphasise that the rule presented here can be mapped to a classical Reinforcement Learning rule in discrete time, namely the Associative Reward Inaction (ARI) [13], [16], [51], see also [12].

### Analysis of System Performance

In the following section, we discuss the system performance with and without lateral connections under four different scenarios focusing on the effects of (i) varying the lateral connection strength, (ii) varying the shape of the reward function, (iii) varying the overlap of the Place Cell receptive fields and (iv) adding uniform noise onto the synaptic updates.

#### Performance and Lateral Connections

**Figure 3 pone-0018539-g003:**
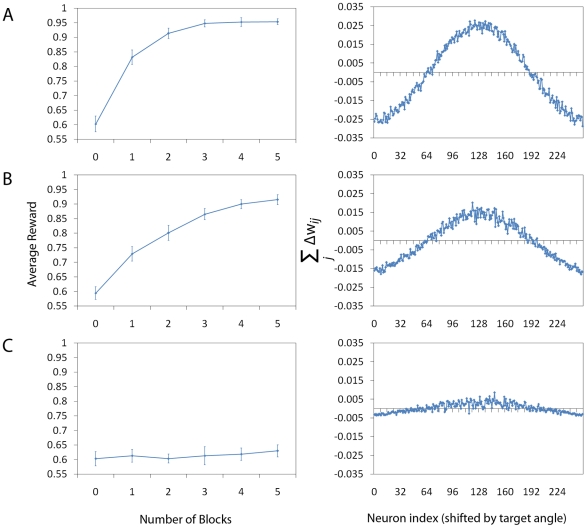
Analysis of System Performance. Panels A–C (left column) show the average performance of 16 animats calculated in the following way. Every animat completes a number of blocks of 512 trials (the number here varies from 0 to 5), with weights being updated at the end of each trial. We term these “blocks of learning trials”. In these figures, 0 blocks of learning trials means that no learning has taken place. The average reward is calculated independently from the blocks of learning trials. Following a block of learning trials, the animat performs of 128 independent (analysis) trials with learning being disabled, based on which the performance of the system is evaluated (mean reward over a total of 128x16 samples). The parameters for systems A–C are the same as in the previous figure (i.e. A: no lateral connections, B: lateral connections and C: very strong lateral connections). We note that the system without lateral connections achieves 70% of reward twice as fast as the system with lateral connections. The system with strong lateral connections completely fails to learn the task. We can obtain a better understanding of the difference between the three systems by plotting the gradient term for each case correspondingly (Panels A–C, right column). We calculate the gradient numerically by summing the value of the potential weight change (before learning where the potential change is maximal) 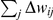 over Place Cell index 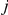 and by shifting the index 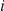 of the Action Cell population so that the peak will always appear at the middle of the graph. To achieve a smooth graph, we average over a total of 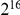 trials. We note that the gradient is larger when lateral connections are absent.

Our simulations show that lateral connections have a strong effect on the performance of the animat. In Figure 2 we show a raster plot of the Action Cell spikes for three different cases. Panel A corresponds to a system without lateral connections (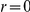), panel B to a system with weak lateral connections (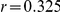) and panel C for a system with strong lateral connections (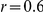). The strength 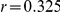 of the lateral connections was chosen as small as possible under the constraint that the dynamics still exhibit a pronounced activity bump (assessed visually). The corresponding performance for these three cases is shown in Figure 3. Panels A–C (left column) show the average performance ( = average reward obtained) over 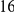 animats as a function of the number of training blocks. Each block consists of 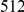 learning cycles. During these blocks, the weights are updated at the end of each trial according to the learning rule of Equation 9. The average reward is calculated independently from the blocks of learning trials. Following a block of learning trials, the animat performs 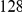 analysis trials with learning disabled, based on which the performance of the system is evaluated (mean reward over a total of 128x16 samples). The sole purpose of this procedure is to obtain an accurate estimate of the performance without interference from learning. Error bars show the standard deviation over the 16 independent animats. Simulation parameters are reported in Table 1. Parameters for neurons are taken from the literature, learning parameters (learning rate 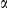, reward baseline 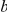) and overlap of neighbouring receptive fields (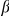) were jointly optimised for each of the systems studied.

**Table 1:** Default model parameters used for producing simulation results.

Parameter	Value	Description
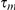	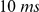	membrane time constant
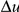	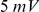	width of the threshold region
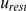	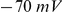	resting potential
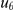	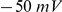	firing threshold
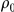	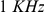	scaling factor of the firing rate
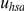	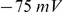	membrane potential after spike emission
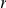	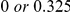	lateral connectivity scale
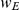	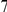	lateral connectivity excitation
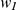	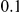	lateral connectivity inhibition
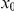	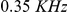	input activity factor
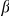	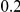	overlap of the receptive fields
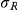	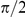	standard deviation of the reward function
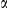	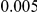	learning rate
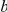	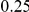	reward baseline
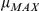	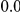	additive noise

Simulation results shown in Figures 1–7 use the above parameters except where indicated otherwise. The value of 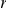 is either 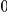 or 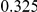 depending on where lateral connections are present (the later indicates that they are). The parameter 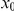 is chosen produce an input of 180Hz. Parameters for the neuronal model are taken from the literature. Other parameters are found through parameter search.

We note that the system without lateral connections achieves the level of 70% of the maximum reward twice as fast as the best of the systems with lateral connections. Furthermore, the system with strong lateral connections completely fails to learn the task. In order to obtain a better understanding of the difference between the three systems, we plot the “weight gradient” 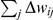 for each case correspondingly (Figure 3, right column). We calculate this gradient numerically in the following way. Before learning (i.e. all weights randomly initialised as zero), we sum up the values of the potential weight changes over the index 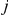 and subsequently shift the index 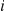 of the Action Cell population by the respective target angle (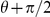) so that the peak will always appear at the middle of the graph. To achieve a smooth graph, we average over a total of 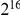 trials. We note that the gradient and hence the information for learning the weights is larger when lateral connections are absent. If lateral connections are strong enough to systematically result in an activity bump, they can achieve at best a speed almost half of the system without lateral connections. We would like to emphasise that the learning rate was optimised for both systems to achieve maximum performance. Increasing further the learning rate for the MHC system would also increase the noise and result in a similar or lower performance. This fact underlines that there is a smaller signal-to-noise ratio in the gradient of the MHC system compared to the system without MHC.

These results can be partially understood by taking into account that in systems with lateral connections, the activity bump and thus the decision settles into a location that is largely determined by the most active neurons over a short time interval at the beginning of the trial. With increasing strength of the lateral connections, this time interval becomes shorter. The neuron models considered here feature escape noise and therefore shorter time intervals result in higher relative noise levels, making systems with strong lateral connections prone to noise (i.e. activity that carries no information about the task). In Reinforcement Learning, performance is strongly affected by the Exploration/Exploitation trade-off, i.e. the percentage of choosing random actions in hope of discovering a “better way” to achieve the goal versus making choices based on the system's current knowledge. Under the conditions discussed here, we may obviously conclude that there is too much noise in systems with strong lateral MHC.

It is worth considering additional aspects that may cause degrading performance in systems with strong lateral connections. The Place Cells have overlapping receptive fields, meaning that one neuron participates in the representation of different states of the system, namely those that are close to its preferred direction. Here closeness is measured in terms of the overlap parameter 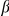. This is a feature of the network that allows information “diffusion” between different learning states and speeds up learning, as simulations (not presented here) show. However, a neuron that participates in representing a state, say 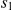, can affect the decision based on the weights it has learned during its participation to another state 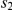. We term this effect “crosstalk”. This phenomenon is more likely to cancel out when the decision of the network is based on the population vector of the activity without lateral connections (corresponding to a linear operation) rather than when a non-linear operation (MHC) is in place.

To collect evidence for this hypothesis, we perform three sets of experiments. In the first one, we vary the shape of the reward function. By making the reward function more sharp, we expect that synaptic weight changes will affect a smaller number of neurons and therefore performance may improve for a system with lateral connections. In the second one, we explicitly increase the overlap of the response function of the Place Cell receptive fields, ie. we increase 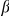, expecting that the system with lateral connections will perform worse. In the third experiment, we add uniform additive noise with a positive bias to the synaptic weight updates, with the intention of “mimicking” the “crosstalk” effect. We expect that performance will deteriorate for the systems with lateral connections more than that of systems without lateral connections.

#### Shape of the Reward Function

**Figure 4 pone-0018539-g004:**
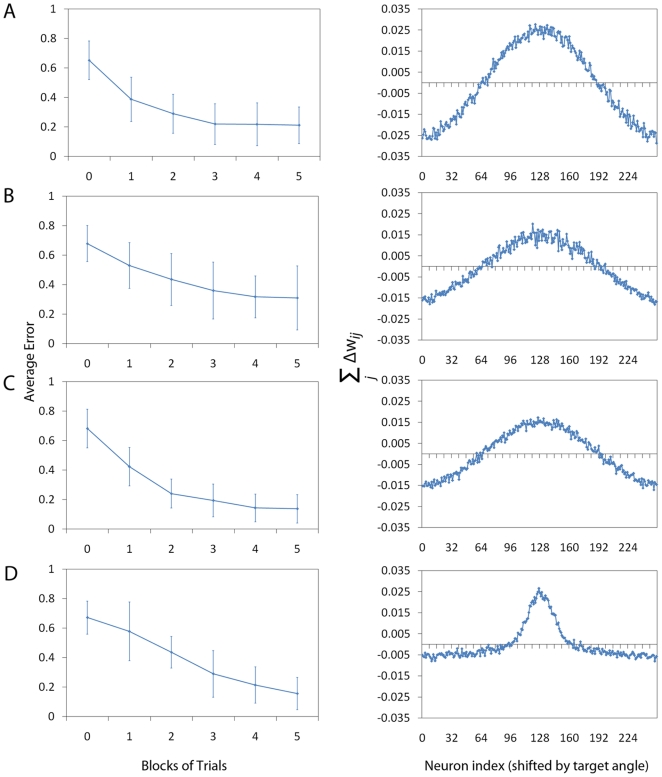
Reward Function Shape. We investigate the effect of the reward function shape by changing it from a “wide” Gaussian (Panels A and B, 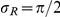) to a “narrow” Gaussian (Panels C and D, 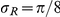). Here we plot average error graphs as they provide a measurement that allows us to compare systems with different reward functions. To produce the average error graphs (panels A–D, left column), we have averaged over 16 independent animats performing 5 blocks of 512 trials. Every point of the graph represents the average normalised error of the system (i.e. the normalised absolute difference between the target angle 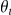 and the decision angle 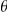) which, similar to Figure 3, is calculated over a separate block of 128 analysis trials (without updating the synaptic weights). Error bars show the standard deviation over the 16 independent animats. We observe that after 5 blocks of trials, the system corresponding to the “wide” Gaussian reward function without lateral connections shown in (A) has reached a lower final error than that of the system with lateral connections (B). When a narrow Gaussian reward is instead used, the system with lateral connections (D) recovers this difference in final error with respect to the system without lateral connections (C). As with the previous plot we show (right column panels) the gradient of the system configurations A–D by plotting the sum of the potential weight change (calculated in the same way as previously). For clarity, plots A and B are repeated from Figure 3. We note that when a narrow Gaussian reward is used the system with lateral connections (D) learns over a very narrow band close to the target angle. In contrast the profile of the system without lateral connections (C) remains consistent with that of the wide reward, learning across a broader range of the population.

We consider the effect of varying the reward function 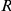 (Equation 8) by modifying the standard deviation of the reward function 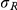 from 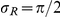 to 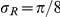. Since our reward is not normalised and in essence by changing 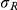 the total amount of reward changes, we introduce an error function 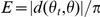, a function of the angle between the decision 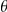 and the target 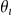, that directly measures system performance. Figure 4 shows the effect of the two different reward functions with respect to the learning performance. Panels A and B show the results for configurations using the “wide” Gaussian with parameters 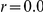 and 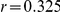 respectively. Panels C and D show the results for configurations using the “narrow” Gaussian with parameters 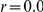 and 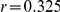 respectively. To produce the average error graphs (left column) in the panels A–D, we have averaged over 16 independent animats performing up to 5 blocks of 512 trials. Every point of the graph represents the average normalised error of the system, which, similar to Figure 3, is calculated over a separate block of 128 analysis trials (without updating the synaptic weights). Error bars show the standard deviation over the 16 independent animats.

We observe that, after 5 blocks of trials, the system without lateral connections (with the “wide” Gaussian reward function) shown in (A) has reached a lower (final) error than that of the system with lateral connections (B). These results are repeated from Figure 3 with a different error measure. With a narrow Gaussian reward function, the system with lateral connections (D) recovers this difference in final error with respect to the system without lateral connections (C). As in Figure 3, we show (right column) the gradient 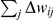 for the system configurations A–D. In summary, systems with strong MHC learn better with more narrow reward functions, whereas systems without MHC achieve the same performance. This is in agreement with our “crosstalk” hypothesis.

#### Overlapping Receptive Fields

We further investigate how increasing the overlap of the Place Cell receptive fields to 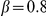 affects the simulations. A higher degree of overlap introduces ambiguity about the position, and a lower performance is anticipated due to the decreased signal to noise ratio in general but nevertheless we expect that this is more observable in the system with lateral connections than the system without.

**Figure 5 pone-0018539-g005:**
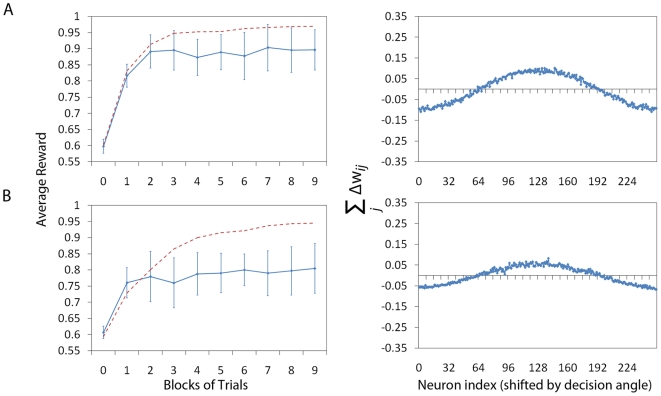
Overlapping receptive fields. Figure shows the effect of increasing the overlap of the receptive fields (to 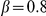) of the Place Cells. Panel A shows a configuration without lateral connections and panel B shows a configuration with lateral connections (and corresponds to systems A and B from Figures 3 and 4). The plots of the average reward (left column, solid line) are calculated in exactly the same way as in Figure 3 shown over 9 blocks of 512 trials, rather than 5. The red dashed line shows the values from Figure 3 over 9 blocks for direct comparison. We can observe that the system without lateral connections is less affected by the increase of the 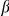 parameter than the system with lateral connections. The plots of the gradient (right column) are produced as in Figures 3 and 4, by plotting the sum of the potential weight change (calculated in the same way as in previous figures).

Figure 5 shows the simulation results. In panel Panel A (left column) we see the average reward for the system without lateral connections versus the blocks of learning trials and in panel B (left column) the reward for the system with lateral connections, again versus the blocks of learning trials. These plots are calculated in exactly the same way as in Figure 3. The red dashed lines shows the corresponding graphs from Figure 3 over 9 blocks of trials for a direct comparison. We can observe that the system without lateral connections is indeed less affected by the increase of the 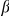 parameter than the system with lateral connections. We also show the gradient (right column panels) similar to Figures 3 and 4, by plotting the sum of the potential weight change. Reduced amplitude of the gradient corresponds to reduced performance.

#### Additive Synaptic Noise

**Figure 6 pone-0018539-g006:**
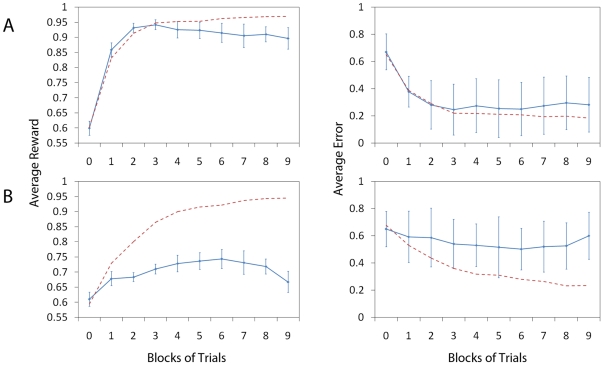
Additive Synaptic Noise. Figure shows the effect of additive uniformly distributed synaptic noise on the network performance by setting 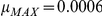 (see Equation 9). Panels A and B show the network performance without and with lateral connections (as in previous figures) respectively. The plots of average reward (left column, solid line) are calculated as in Figures 3 and 5 showing learning curves over 9 blocks of 512 trials. The red dashed line shows the values without noise from Figure 3 (systems A and B correspond) for direct comparison. Similarly, the plots of average error (right column, solid line) are calculated as in Figure 4. The red dashed line shows the values without noise from Figure 4 (again, systems A and B respectively). We can observe that both the average reward and average error performance measures show that the system without lateral connections is far more robust to noise applied directly to the synaptic weight.

Finally, we heuristically mimic the effect of “crosstalk” on the synaptic connections by adding noise with a positive bias to the weight update rule described by equation 9. The non-zero bias is motivated by the fact that the shape of the gradient indicates that learning takes place mostly via positive synaptic updates. To find the appropriate parameter regime for the noise, we have performed a parameter search over the variable 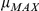 Figure 6 shows the simulation results for 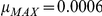. Panels A and B show the network performance without and with lateral connections (as in previous figures) respectively. The plots of average reward (left column) are calculated as in Figures 3 and 5. The red dashed line shows the values without noise from Figure 3 (systems A and B correspond) for direct comparison. The plots of average error (right column) are calculated as in Figure 4. The red dashed line shows the values without noise from Figure 4 (again, systems A and B correspondingly). As expected, we observe that both the average reward and average error performance measures show that the system without lateral connections is more robust to noise added directly to the synaptic weight updates.

**Figure 7:**
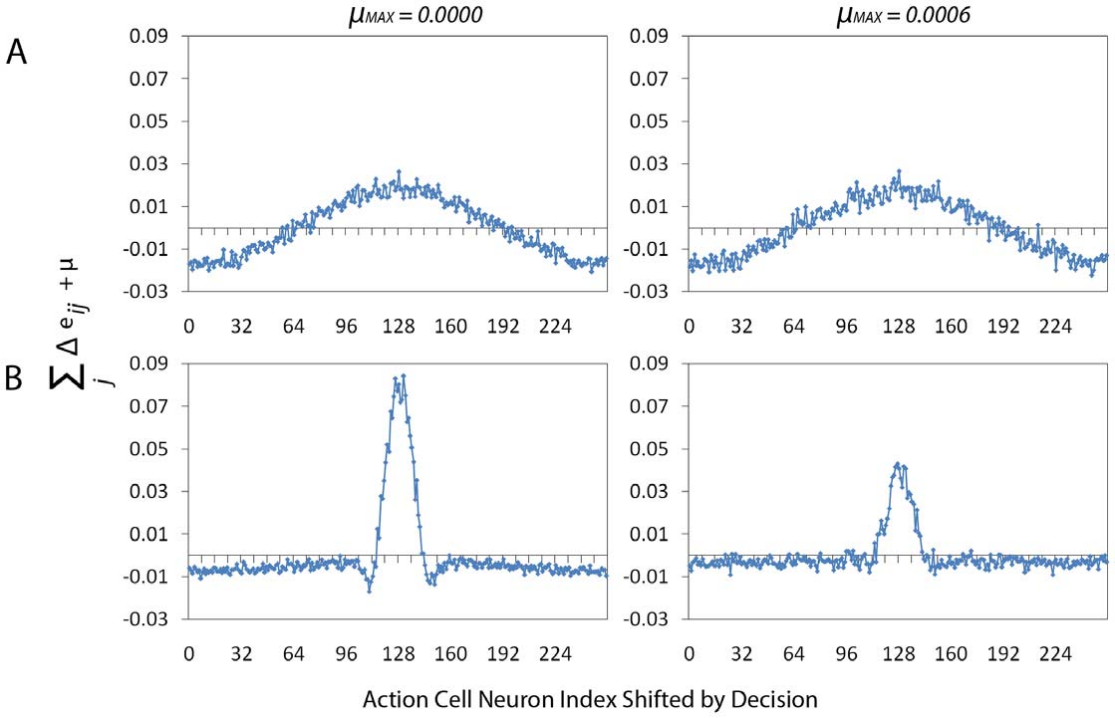
Noise Analysis. To obtain a better understanding of the difference between the performance of the two systems from Figure 6 (A: no lateral connections, B: with lateral connections) we plotting the eligibility trace for each case with and without an additive noise term 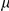. This corresponds to 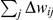 from Equation 9 for 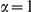 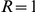 and 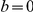 and allows us to look at the gradient information without taking into account the shape of reward. We calculate the eligibility trace (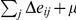) numerically by summing the value of the potential eligibility trace (before learning, where the potential change is maximal) over Place Cell index 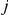 and by shifting the index 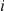 of the Action Cell population so that the maximum will be at the middle of the graph. To obtain smooth curves, we calculate this value over a total of 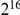 trials. The left column panels show the eligibility trace without noise. The right column panels show the the eligibility trace, including noise (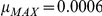 as in Figure 6). In both cases the same random seeds are used when generating spikes and target angles to ensure both systems are presented with the same information. The resulting right column figures therefore give an indication of the effect of the noise. We note that the eligibility trace of system without lateral connections is relatively unchanged by the effect of noise, where as the system with lateral connections results in an eligibility trace drastically reduced in magnitude.

We further analyse the difference between the performance of the two systems in Figure 7 (A: no lateral connections, B: with lateral connections) in the following way. We plot the eligibility trace for each case with and without an additive noise term 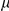. This corresponds to 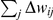 from Equation 9 for 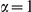 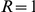 and 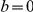 and allows us to look at the gradient information without taking into account the shape of the reward function. We calculate the eligibility trace (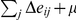) numerically by summing the value of the potential eligibility trace (before learning where the potential change is maximal) over the (input neurons) index 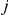 and by shifting the index 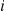 of the Action Cell population so that the maximum will be at the middle of the graph. Curves resulting from this procedure are particularly noisy when a small number of samples is used. To smooth them out we calculate them over a total of 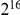 trials. The left column panels show the eligibility trace without noise. The right column plots show the eligibility trace, including noise (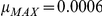 as in Figure 6). We note that the eligibility trace of the system without lateral connections is relatively unchanged by the effect of noise, where as the system with lateral connections has an eligibility trace which is drastically reduced in magnitude.

## Discussion

Here, we presented a study of two spiking networks, one with lateral connections among the neurons of the output layer (Mexican Hat-type connectivity, MHC) and one without lateral connections. The two networks are learning a simple association learning task, which corresponds to a simplified yet self contained version of a full navigation problem. We use GPU programming to perform exhaustive optimisation of the simulation parameters and to produce a set of scenarios that investigate the learning performances of the two systems under various conditions. We conclude the following. In systems that feature lateral MHC, which introduces a non-linear (“non-democratic”) decision making process, the first few spikes occurring in each trial can significantly influence the activity bump formation and therefore the decision, see also [7]. This effect manifests itself in a low signal-to-noise ratio in the learning rule compared to systems without lateral MHC, which was revealed by investigating the weight “gradient” 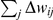. As a result, more samples are required for MHC systems to reliably extract the values for the synaptic weights that correspond to the “correct” input-output relationship. In the extreme case of strong lateral connections, the activity bump formation of the recurrent network (the “decision”) is strongly driven by noise rather than the feed forward input and thus no reasonable weights are learned. If lateral synaptic weights are present, and strong enough to result in bump formation, the system can at best reach half of the learning speed of a network without MHC.

Furthermore, we formulated the following additional hypothesis that may partially explain the reduced learning performance in MHC systems. Our simulation results hint that systems with MHC are prone to “crosstalk” between learning trials when the state of the agent is coded with the help of neurons with opverlapping receptive fields. We borrow the term “crosstalk” from electronics to describe that a neuron participates in different “circuits” or sub-networks (consisting of connections from active input neurons to output neurons). Learning in one sub-network may lead to synaptic changes that could affect another sub-network. We identify this behaviour by performing three additional simulations:

1. Increasing overlap of the receptive fields (state representation),
2. Widening of the rewarded function and
3. Adding uniform noise with a positive bias to the weight vector.

In all these cases, learning is impaired for the “non-democratic” decision making in MHC networks. This can be understood in the following way. Neuron 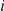 (Place Cell) that participates in the representation of state 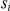 may have a weak connection to the neurons (Action Cells) participating to the “correct” decision 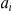. However, it may have a strong connection to neurons encoding a different action 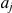 acquired during its participation to representing 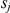. As a consequence, when being active again during 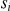, it could bias the decision to a wrong action. This “crosstalk” is of course also present in the system without lateral MHC. There, however, crosstalk effects tend to cancel out due to the linear population vector readout and the symmetry of the task. This is in contrast to MHC systems where the crosstalk effects do not cancel out in general due to the non-linear dynamics of the activity bump formation. This results in further reduction of the signal-to-noise ratio of the learning rule for MHC systems, and as a consequence learning slows down and converges to lower performances. Hence “democratic” decision making is more robust than the “non-democratic” alternative.

We would like to emphasise that the presence of noise in neural systems is not necessarily a curse. Though in our study noise seems to impair a network with MHC, exploration itself is an essential part of Reinforcement Learning. Moreover, noise in neural systems might be beneficial as, for instance, it prevents synchronisation and allows for fast and accurate propagation of signals, see [52]–[54].

As a final note, the study here would have been very difficult using a low cost desktop computer, without resorting to GPU programming. Not only parameter search itself can be very time consuming but producing statistically accurate results for systems with multiple sources of noise (poisson neurons, escape noise, additive noise) can be very challenging. To achieve smooth graphs, averages were often calculated over 2,048 independent trials. This was possible due to exploiting the parallelism of the GPU architecture by running multiple instances of the model in parallel. We hope that our **Methods** will be applicable to problems of similar nature, whenever numerous samples are required to guarantee the validity of the results.

## Methods

This section describes the GPU implementation of the model presented in **Results** and is divided in three parts. The first part introduces the CUDA programming Application Programming Interface (API) and hardware architecture. The second part describes our implementation and the third part evaluates the performance of our GPU implementation and suggests techniques which will be used in the future to further improve simulation performance. A general introduction to GPU computing and CUDA can be found at [55].

### An Introduction to the GPU and CUDA

We have chosen to implement our spiking neural network on the GPU using the CUDA programming API which is described in detail in the CUDA Programming Guide [56]. The key concept within this API is the use of a special purpose function (a “kernel” function) identifying code to be run on the GPU device and which describes the behaviour of a large number of independent but functionally equivalent units of computational execution. Each independent unit of execution, more commonly referred to as a thread, can be assumed to operate simultaneously (i.e. in parallel) but on a different stream of data. This differs from traditional CPU parallelism in that the parallelism is formed through the distribution of the streams of data across processors, referred to as data parallelism, rather than of separate tasks, which are described by different functions, known as task parallelism. In Computational Neuroscience a simple conceptual example is the following. A non-recurrent population of similar neurons can be simulated using the data parallel thread paradigm as each individual neuron is described by the same functional behaviour (i.e. the kernel function) but operates in parallel using its own local stream of information which indicate, for example, the neurons membrane potential, synaptic weights, etc.

At a hardware level the CUDA architecture consists of a varying number of multiprocessors each with access to dedicated Dynamic Random Access Memory (DRAM). The DRAM is equivalent but independent from traditional memory accessed by the CPU and therefore information on the CPU (host) must be transferred to the GPU (device) memory before any execution of a kernel can take place (and vice versa once GPU execution has completed). Each multiprocessor contains an array of scalar processors, responsible for execution of the individual threads. A scalar processor is the simplest form of a processor that processes one data point at a time. Large groups of threads are split between physical multiprocessors by arranging them into smaller groups which are called thread blocks. For the sake of simplicity one can assume a simple mapping between each scalar processor and individual threads. For a more factual explanation of the reality of technical aspects such how threads are broken down into smaller executional units and how threads are interleaved on a single processors the reader is directed towards the CUDA programming Guide [56] or the book CUDA by Example [55].

Perhaps the most powerful feature of the CUDA architecture is the availability of a small amount of user configurable shared memory cache, which is an area of memory available on each multiprocessor considerably faster to access than main memory, that allows simple communication between threads within the same group. Considering the simple conceptual neuron example presented above it is not immediately clear how this can be of any benefit. To illustrate the benefits of the memory cache consider a matrix multiplication example where each thread is responsible for computing an element 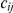 of the Matrix C which is the product of two matrices A and B. Naively each thread may compute 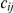 by considering the dot product of row 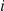 and column 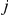, an operation which requires each thread to read both an entire column and row from matrices A and B respectively. Alternatively by using the memory cache intelligently, the total number of memory reads from the two matrices can be drastically reduced (and performance increased substantially) by allowing each thread within a group to calculate a single product. Each of these single products can be stored within the cache and then used by the other threads in calculating the element 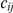 of C. The example assumes a situation where a single block of threads is small enough to hold the single element products without exceeding the size of the memory cache. More generally, it is advisable to maximise the ratio of computational arithmetic to memory access, commonly referred to as arithmetic intensity, by maximising usage of the user configurable cache (or other similar caches which are not discussed within this paper) which will ensure simulations attain maximum performance.

### GPU programming in Neuroscience

Prior to our own work there has already been some interest in performing simulations of neuron populations on high performance architectures including GPUs. Simulations of artificial neural networks in discrete time [57], [58] present the most simplistic case where large matrix multiplication operations, known to be very efficient on the GPU, bear the brunt of the computational load. With respect to simulating more biologically plausible neuron systems operating in continuous time and where dense matrix multiplications can not be used, a notable GPU implementation is that of Bernhard and Keriven [59] which employs the technique (prior to modern CUDA and OpenCL libraries) of mapping an algorithm to graphics primitives in order to utilise GPUs. A multi-purpose spiking neural-network simulator is implemented, using a limited neighbourhood of connections between local neurons to minimise communication overhead. The Brian simulation package [60], [61] not only provides a simple syntax for specifying systems of spiking neurons within a Python Application Programming Interface (API) but also demonstrates GPU acceleration for various aspects of the simulation processes. The first use of CUDA was in the context of accelerating the automatic fitting of neuron models to electro-physiological recordings [62]. More recently [63] the same authors have turned their attention to GPU accelerated simulations of neuron dynamics, performing simulations of a number of spiking neuron models on GPUs. However, the simulation of propagation and back-propagation of spikes is not implemented on the GPU but instead it remains on the Central Processing Unit (CPU) due to the lack of inherent parallelism. An alternative configurable simulation environment for simulating spiking neurons on the GPU is proposed in [64]. Moreover, Bhuiyan et al. [65] have demonstrated the implementation of the Hodgkin Huxley and Izhikevich models of neurons on a number of parallel architectures including multi-core CPUs, the Cell Broadband Engine and the GPU. In their work, the GPU is used to perform the simulation of a single layer of neurons, whereas the simulation of the second layer of neurons and processing of spike data takes place on the host (after the data has been transferred from the GPU device). This work shows that the GPU is able to perform considerably well for the Hodgkin Huxley model, with a speed-up of over 100 times for 5.8 million neurons. This is attributed to the ratio of arithmetic work load over data transfer (also known as arithmetic intensity) which is considerably greater for the Hodgkin Huxley model than for the Izhikevich's model, for which the speed-up (only being 9.5) does not amortize the cost of communication between neurons. Aside from simulations of neuron systems using commodity (or consumer hardware), several research projects aim to perform large scale simulations of biological neuron systems using large computer clusters. The Swiss institution EPFL are currently developing highly accurate sub-neuron level simulations [66] and the IBM Almaden Research Center has simulated systems of sizes equivalent to that of the cortical system of a cat [67], both using the IBM BlueGene/L supercomputing architecture. Additionally, the Biologically Inspired Massively Parallel Architecture (BIMPA) project takes a completely new approach to brain simulation choosing to design a highly interconnected low power hardware architecture inspired by neuron interconnectivity [68]. Another similar attempt, the Fast Analog Computing with Emergent Transient States (FACETS) project resorted to dedicated analogue VLSI hardware [69]–[71] to achieve high simulation speeds and developed PyNN [72]–[77], a Python-based simulator-independent language for building neuronal network models.

### Implementing our Spiking Neuron Model in CUDA

**Figure 8:**
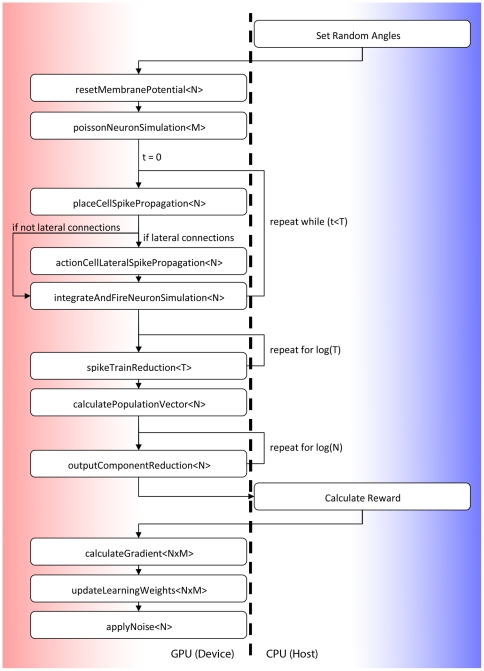
Simulation Flowchart. Figure shows the simulation process of our spiking neural network model. Steps shown on the left are broken down into CUDA kernels where the figure in 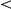
brackets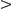 represents the total number of threads which are invoked for each kernel (for simplification this assumes only a single independent trial). The value N represents the number of Action Cell neurons (256), the value M represents the number of Place Cell neurons (256) and the value T represents the total number of number of discrete time steps, i.e. 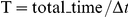 (which is 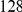 when 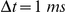). Steps shown on the right indicate calculations performed on the Host CPU.

In our implementation, we discretize the model's equations using Euler's method. Conceptually the various stages of a single trial of the learning are broken down into small functional units which map directly to CUDA kernels. Figure 8 shows these various kernel functions (running on the GPU device) as well as the CPU host functions which are described individually below in more detail.

While the figure refers to a simple case where only a single trial is considered, in reality we are able to perform many independent trials at once. Each of these independent trials may use a different parameter configuration or the same configuration. In addition to the main simulation loop, additional host side code is used to allocate all GPU memory in advance and seed a number of independent streams of pseudo random parallel numbers [78] which are used in spike generation and noise application. Each of the kernels described below uses a common technique called memory coalescing which describes the processes of reading sequential consecutive (in each thread) values from memory in a way which allows fewer large memory requests to be issued, improving performance.

#### Set Random Initial Angles

Preceding the brunt of the GPU simulation the CPU host calculates a random unique initial animat location (angle) for each trial to be performed. This is then transferred to the GPU using a CPU host to GPU device memory copy. As the number of possible initial locations is relatively small, it does not make sense to perform this operation on the GPU due to overheads associated with calling a CUDA kernel function.

#### Reset Membrane Potential Kernel

This CUDA kernel is responsible only for resetting the block of GPU memory allocated to hold the N Action Cell membrane potentials from the previous learn step's final value to 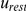.

#### Poisson Neuron Simulation Kernel

This CUDA kernel is launched with M total threads each of which calculates the firing rate frequency (given in Equation 1) for each of the M Place Cells. This value is then compared to T GPU generated random numbers, where T is the number of discrete steps in the total time period (i.e. 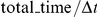), to output T total spike values of 0 (not fired) or 1 (fired). Conceptually it would have been possible to launch M*T independent threads to perform this operation however the overhead of recalculating the firing rate frequency for each thread is greater than the overhead of performing a loop over T.

#### Place Cell Spike Propagation Kernel

This CUDA kernel represents the first stage of the simulation loop over time T. For each time step the kernel is launched and performs a dense matrix vector multiplication for the Place Cell inputs at time 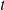 and the corresponding synaptic strengths for each Place Cell to Action Cell combination. Our implementation of the matrix vector multiplication is based upon the technique described by [79] and uses shared memory to cache the input spikes and an optimisation called loop unrolling (see [56] or [55]) to perform a parallel reduction to reduce instruction overhead.

#### Action Cell Lateral Spike Propagation Kernel

As with the “placeCellSpikePropagation” kernel, this CUDA kernel performs a dense matrix vector multiplication for the Action Cell output spikes from the previous time step (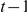) and the corresponding lateral cell synaptic connection weights.

#### Integrate and Fire Neuron Simulation Kernel

**Figure 9:**
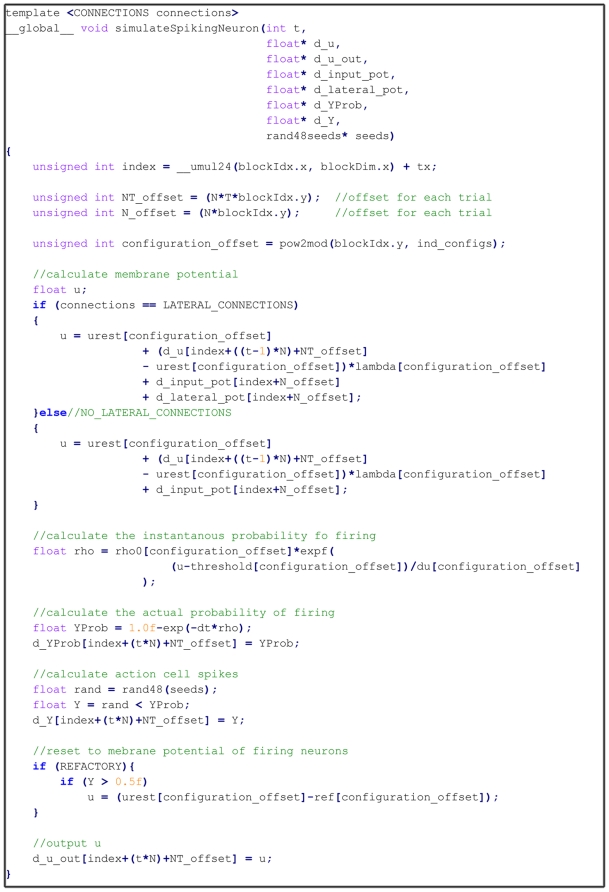
Spiking Simulation Kernel. This figure shows the CUDA kernel used to perform the update of a spiking Action Cell neuron. Conceptually the kernel is relatively simple, using linear algebra style computation to update the neurons membrane potential (d_u_out) using the previous potential (d_u). The kernel also saves to the GPU Device memory the probability of firing (d_YProb) and the actual spike contribution (d_Y) of the neuron without requiring any localised caching of data. The variable index is calculated using a thread (tx) and block of thread identifiers (blockIdx.x) and represents the neuron index position within a large list of all neurons being simulated by the kernel. The value blockIdx.y indicates the independent trial number for each neuron this used to calculate the offset variables of N_offset and NT_offset which are used to ensure unique values for each neuron in each independent trial are accessed. The variable configuration_offset is similarly used to represent an index in which to look up one of the independent parameter configuration values. The corresponding function pow2mod uses bit shift operations to perform an efficient integer modulus operation where the divisor is a power of 2. The kernel function arguments are all prefixed with “d_” to indicate memory on the GPU device rather than the GPU host. The argument “seeds” is also an area of memory on the GPU device which is used to hold seeds for the parallel random number generation.

This CUDA kernel launches a thread for each of the N Action Cells, each of which updates and saves to global memory the membrane potential using the spiking activity contributions of the Place Cell spike propagation (and the Action Cell lateral spike propagations if the system uses lateral connections). Following this, the kernel computes the instantaneous probability of firing (which is output to global memory) and then using a randomly generated number outputs a spiking value at the given discrete time step 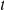. For neurons which produce a spike emission, a refractory period is applied by resetting the membrane potential to the value 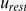. Figure 9 shows our actual CUDA kernel code for performing this stage of the simulation.

#### Spike Train Reduction Kernel

In order to calculate the population vector of our Action Cells we consider the average firing rate over the total time period of 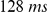. To calculate this quantity in parallel on the GPU we sum the number of spikes of each neuron over the total number of discrete time steps T (i.e. 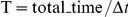) by performing a parallel reduction of values over 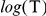 steps. We use the parallel reduction technique described by [80] which employs a number of advanced optimisation techniques to obtain maximum performance. Before this kernel operates we also perform a matrix transpose of the spike train information Y to ensure that the reduction step is able to perform coalesced memory reading. The additional computation proves more efficient than performing the reduction using an uncoalesced memory access pattern.

#### Calculate Population Vector Kernel

This CUDA kernel calculates the output components of the population vector. That is the individual terms 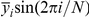 and 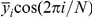 for each Action Cell 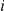 from Equation 6.

#### Output Component Reduction Kernel

The population vector is calculated by performing two parallel reduction operations on the output components in order to reduce them to a pair of single values. The same parallel reduction technique is used as in the “spikeTrainReduction” step, however the output components of the population vector do not need to be transposed to ensure a coalesced memory access pattern is followed. Whilst there is a large overhead for performing this operation with very few threads in the final 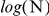 steps this is compensated for when multiple independent trials perform the same kernel function.

#### Calculate Reward

The calculation of the reward function is not suitable for GPU implementation as it requires calculation of only a single value per trial. Fortunately, as the output compensates have been reduced to two single values in the previous step, the transfer of data from the device back to the host is minimal (only two values). Following the calculation of the reward function as described by Equation 8, the reward is then copied back to the device to be used in subsequent steps.

#### Calculate Gradient Kernel

This kernel performs the calculation of the term 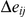 from Equation 9. Functionally this operation is a dense matrix multiply operation based on the CUDA matrix multiplication example from the CUDA SDK. This uses shared memory to substantially reduce the number of global memory reads. The matrix P (the probability of firing) is transposed to ensure coalesced memory access. Spike train Y was transposed and stored previously (during the “spikeTrainReduction” step).

#### Update Learning Weights Kernel

This CUDA kernel performs the synaptic weight adjustment of the connections between the Action Cells and Place Cells. They are updated using Equation 9 where the reward value and the component of the gradient for each Action Cell 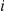 and Place Cell 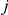 combination are multiplied.

#### Apply Noise Kernel

This CUDA kernel is called only if the noise contribution value 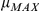 is greater than 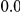. The kernel simply applies a basic linear operation to add a noise component to each synaptic strength connection value. This is performed using M threads which loop over the N synaptic weights. While it would be possible to perform this with M*N independent threads (and as part of the previous step) this would require a considerably larger number of independent random streams (a separate stream is required also for each independent trials) which we have found to cause the random number generation algorithm to break down (at roughly 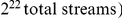.

Simulation code and analysis scripts, developed in CUDA, are available from ModelDB [81] at http://senselab.med.yale.edu/modeldb via accession number 136807. In the results presented here we use a time step of 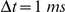, but we have verified that our results remain consistent when smaller time steps are used.

### Performance Analysis and Discussion

**Figure 10:**
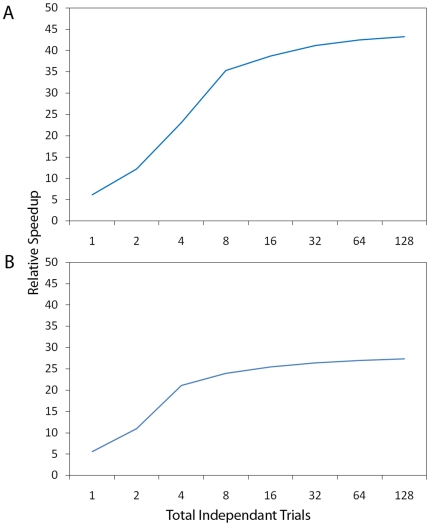
Relative Performance Speedup. Figure shows the relative performance improvement of our GPU model with respect to a similar Python implementation using numpy's BLAS implementation. Relative performance refers to the percentage increase in performance by considering the absolute timings of the two implementations over an entire simulation. The horizontal axis indicates **Ind_total**, that is the total number of independent trials which in this case represents independent animats (rather than animats in different configurations). A: Represents the case for our model without lateral connections. B: Represents a case with lateral connections which is the same however it includes an additional code execution to perform simulation of the lateral spike propagation.

We consider the performance of our GPU implementation by comparing its performance to that of a version of the same model implemented in Python using the BLAS enabled numpy library. This library uses natively implemented SSE vector instructions (which optimise operations carried out on groups of data) and multi-core support to accelerate linear algebra operations. Figure 10 shows the performance of the system configuration without and with lateral connections (the difference being the execution of the “actionCellLateralSpikePropagation” function). Performance is measured in relative speedup over the Python implementation by considering the difference in execution time between the two versions over the period of an entire simulation 10 blocks of 512 trials (10 blocks of 256 for the system without dynamics) including analysis of 128 trials for each of the 10 blocks. The hardware configuration used for benchmarking consists of a Intel i7-930 quad core CPU with 6 GB of RAM and a NVIDIA GTX 480 GPU. We have observed that as the number of independent trials is increased, the relative performance does also. This is not surprising as with a single independent trial the number of threads used by many of CUDA kernels is far below the number required to ensure all of the hardware multiprocessors have work to carry out. We have found that 8 independent trials to be the minimum to ensure that each of the multiprocessors will be kept busy. Beyond this number of independent trials, performance levels out for each of the two system configurations. This is useful as we have found that a minimum of 8 independent trials (we have used 16 throughout our experimentation unless otherwise stated) provides a good numeric numeric average to calculate the standard deviation of the average reward value for each learning step. For each of the simulation result obtained in Figure 10 we have used an optimal value of 256 threads per block. By ensuring we only use system wide model parameter values (i.e. N, M and T) of power two numbers we also ensure that our kernel launches fit neatly within our chosen thread block size. Whilst it would be possible to use non power of two values the decision to use them represents the best case scenario for the GPU as their is exactly the number of threads required to perform the computations.

**Figure 11:**
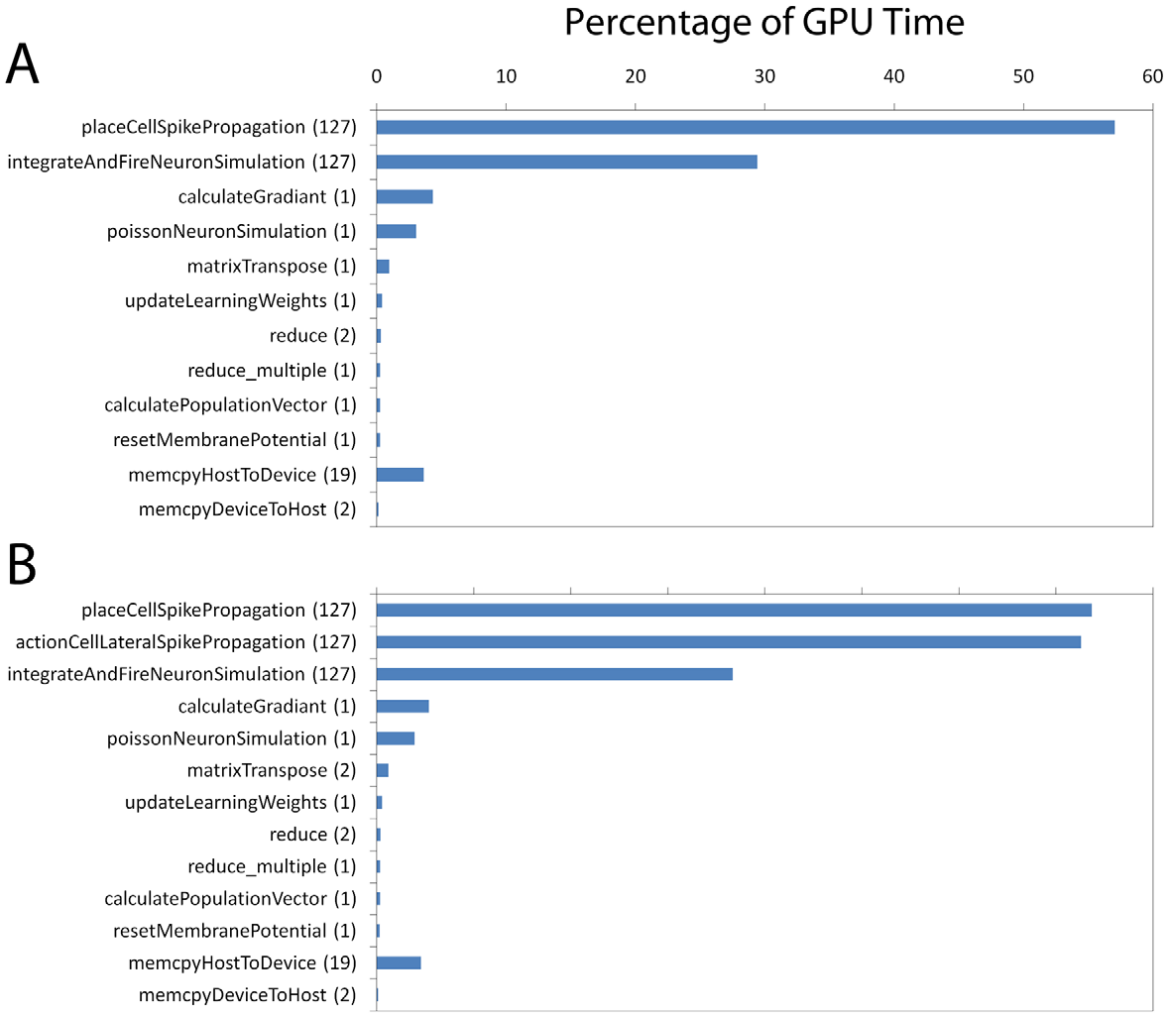
Performance Profile. Figure shows the performance profile of our GPU implementation with respect to where GPU time is spent during the simulation (shown as a percentage for each GPU kernel corresponding with figure 8). The figure in brackets next to the vertical axis label indicates the total number of times the kernel function is called over single “learn step” with a total time simulation period of T = 128 and 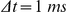. An additional amount of CPU time is also required however this is negligible in the scale of the overall simulation. A: Represents the case for our model without lateral connections. B: Represents a case with lateral connections which is the same however it includes a kernel function “actionCellLateralSpikePropagation” which performs the lateral spike propagation simulation.

The performance difference between the two system configurations in Figure 10 can be accounted for by considering where the majority of GPU time is spent. Figure 11 shows where the percentage of GPU simulation time is spent for both system configurations (without and with lateral connections). The obvious observation is that for both systems the majority of simulation time is spent performing the spike propagation operations which are repeated 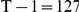 times during our profiling observation. In the case of the system with lateral connections this is most evident and a total of only 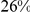 constitutes other aspects of the simulation (in contrast with 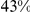 where there are no lateral connections). As all other aspects of the system simulation are the same it can be deduced that the calculation of spike propagations is considerably less efficient than other aspects of our implementation. This is not surprising as each propagation calculation is a matrix vector operation which is inherently bound by memory operations (bandwidth) is is therefore less likely to offer the kind of performance improvement we can observe with operations which contain a higher percentage of arithmetic intensity (such as the “integrateAndFireNeuronSimulation” kernel) or heavy use of shared memory (such as the gradient calculation). As a result we must conclude that whilst our simulation performance is considerably better when using the GPU (a simulation without lateral connections and with 128 independent trials takes for example almost 7 hours on the CPU when compared to 9 minutes on the GPU) that future work must address the limitation of the matrix vector performance. In previous work [59], which used simulated spiking neurons for pattern recognition tasks, this has been done by considering only a small subset of feed forward connections (based on a 2D grid layout). For more general neuron populations using all to all feed forward connections, we propose that future work will assess the use of a sparse vector matrix implementation which will reduce computational load (and increase performance) by performing calculations only where there are spikes (perhaps by reducing the matrix/vector magnitudes using parallel reduction). Alternatively we may consider the use of an agent based simulation framework on the GPU (such as GPU FLAME [82], [83]) which will similarly avoid redundant computations by processing spikes as communicated messages passed between neurons rather than through the dense matrix based mathematical implementation presented here. The vector containing spike information is sparse in the sense that over a single discrete time step only a small number of neurons actually fire. As a result the matrix vector multiplications, which are where the majority of GPU time is spent, are inefficient as they are performing a dense calculation i.e. calculating many terms with zero values. Using a sparse matrix vector operation would make this more efficient as would using an agent based approach where neurons would perform computation only on spikes which were generated.
